# Expression Analysis of LEDGF/p75, APOBEC3G, TRIM5alpha, and Tetherin in a Senegalese Cohort of HIV-1-Exposed Seronegative Individuals

**DOI:** 10.1371/journal.pone.0033934

**Published:** 2012-03-27

**Authors:** Kim Mous, Wim Jennes, Makhtar Camara, Moussa Seydi, Géraldine Daneau, Souleymane Mboup, Luc Kestens, Xaveer Van Ostade

**Affiliations:** 1 Laboratory for Proteinscience, Proteomics and Epigenetic Signaling, Department of Biomedical Sciences, University of Antwerp, Wilrijk, Belgium; 2 Laboratory of Immunology, Department of Microbiology, Institute of Tropical Medicine, Antwerp, Belgium; 3 Laboratory of Immunology, Department of Bacteriology-Virology, Centre Hospitalier Universitaire Le Dantec, Cheikh Anta Diop University, Dakar, Senegal; 4 Department of Infectious Diseases, Centre Hospitalier Universitaire Fann, Cheikh Anta Diop University, Dakar, Senegal; University of Toronto, Canada

## Abstract

**Background:**

HIV-1 replication depends on a delicate balance between cellular co-factors and antiviral restriction factors. Lens epithelium-derived growth factor (LEDGF/p75) benefits HIV, whereas apolipoprotein B mRNA-editing catalytic polypeptide-like 3G (APOBEC3G), tripartite motif 5alpha (TRIM5α), and tetherin exert anti-HIV activity. Expression levels of these proteins possibly contribute to HIV-1 resistance in HIV-1-exposed populations.

**Methodology/Principal Findings:**

We used real-time PCR and flow cytometry to study mRNA and protein levels respectively in PBMC and PBMC subsets. We observed significantly reduced LEDGF/p75 protein levels in CD4+ lymphocytes of HIV-1-exposed seronegative subjects relative to healthy controls, whereas we found no differences in APOBEC3G, TRIM5α, or tetherin expression. Untreated HIV-1-infected patients generally expressed higher mRNA and protein levels than healthy controls. Increased tetherin levels, in particular, correlated with markers of disease progression: directly with the viral load and T cell activation and inversely with the CD4 count.

**Conclusions/Significance:**

Our data suggest that reduced LEDGF/p75 levels may play a role in resistance to HIV-1 infection, while increased tetherin levels could be a marker of advanced HIV disease. Host factors that influence HIV-1 infection and disease could be important targets for new antiviral therapies.

## Introduction

Human immunodeficiency virus type 1 (HIV-1) interacts with many cellular host proteins during its replication cycle [1]–[3]. Some of these proteins are required for HIV-1 replication while others exhibit antiviral activity. Lens epithelium-derived growth factor p75 (LEDGF/p75) [4] is a cellular co-factor, whereas apolipoprotein B mRNA-editing catalytic polypeptide-like 3G (APOBEC3G, A3G) [5], tripartite motif 5alpha (TRIM5α) [6], and tetherin (BST2, CD317, HM1.24) [7] are cellular factors with distinct antiviral activities. The main working mechanisms of these factors can be summarized as follows. LEDGF/p75 assists in the integration of viral cDNA into specific regions of the host's genetic material [8], [9]. APOBEC3G inhibits HIV replication by inducing G-to-A hypermutation of the viral HIV-1 genome during reverse transcription [5], [10]. TRIM5α structurally disorders the retroviral capsid, leading to the interruption of the natural “uncoating” process in a species-specific manner [11], [12]; and was recently described to promote innate immune signaling [13]. Tetherin, finally, inhibits HIV release by tethering newly formed retrovirus particles to the cell membrane [7].

The significance of these four proteins as possible correlates of protection against HIV-1 remains to be determined. Several studies on peripheral blood mononuclear cells (PBMC) have found that APOBEC3G mRNA levels correlate positively with the CD4 count and negatively with the viral load of untreated HIV-1-infected subjects [14]–[16], suggesting that APOBEC3G contributes to the control of HIV-1 infection. However, other studies did not find such correlations for APOBEC3G [17]–[19] or TRIM5α [20] mRNA. In addition, certain studies compared the expression of APOBEC3G or TRIM5α between distinct study populations like untreated HIV-1-infected subjects, healthy controls, and HIV-1-exposed seronegative individuals. Some studies observed lower mRNA expression levels of APOBEC3G [14], [17], [19] or TRIM5α [20] in untreated HIV-1-infected subjects compared to healthy controls, although the opposite was also found for APOBEC3G [15]. APOBEC3G mRNA and protein expression levels were also studied respectively in PBMC and PBMC-subsets (CD4+ and CD8+ lymphocytes, and CD14+ monocytes) of HIV-1-exposed seronegative subjects relative to healthy controls [16], [19], [21]. Here too, conflicting data were obtained, showing either similar [19] or higher [16], [21] levels of antiviral APOBEC3G in HIV-1-exposed seronegative subjects relative to healthy controls.

In the present study, we investigated mRNA and protein expression levels of the 4 HIV-1-related factors LEDGF/p75, APOBEC3G, TRIM5α, and tetherin in HIV-1-exposed seronegative subjects, healthy controls, untreated HIV-1-infected subjects, and antiretroviral therapy-treated HIV-1-infected subjects. HIV-1-exposed seronegative subjects are individuals who remain HIV-1 seronegative despite frequent and unprotected exposure to HIV, and are observed in cohorts of commercial sex workers, men having sex with men, intravenous drug users, and discordant couples [22]. In this study, HIV-1-exposed seronegative subjects were enrolled from a Senegalese cohort of HIV-1 serodiscordant couples. Real-time quantitative polymerase chain reaction (qPCR) was applied to study mRNA expression in PBMC. Protein expression levels were determined simultaneously in different PBMC subsets by flow cytometry. We hypothesized that expression levels of the above-mentioned proteins may contribute to the putative *in vivo* HIV-1 restriction in HIV-1-exposed seronegative subjects.

## Materials and Methods

### Ethics Statement

The study was approved by the Internal Review Board of the Institute of Tropical Medicine (Antwerp, Belgium) and by the Ethical Committees of the Senegalese Ministry of Health (Dakar, Senegal) and the University Hospital of Antwerp (Belgium). All study subjects gave written informed consent prior to enrollment.

### Study population

This study was conducted on a cohort of heterosexual couples recruited at the Department of Infectious Diseases at the Fann University Teaching Hospital, Dakar, Senegal, as previously described [23]. Twenty three HIV-1 exposed seronegative subjects (HESN) in HIV-1 serodiscordant couples, 23 healthy controls (HC) in HIV-1-negative seroconcordant monogamous couples, and 45 HIV-1-infected patients in HIV-1 serodiscordant or HIV-1-positive seroconcordant couples were studied. Within the group of 45 HIV-1-infected patients, 9 patients were untreated HIV-1-infected subjects (HIV-UT) and 36 patients were antiretroviral therapy-treated HIV-1-infected subjects (HIV-ART). All couples had a sexual relationship of at least 7.5 years. HESN and HC were matched for gender. Blood samples and standard questionnaires with information on socio-demographics and sexual behavior were collected. Male condom provision and sexual risk reduction counseling were provided during every visit.

### Sample collection and processing

Whole blood samples were collected in EDTA tubes. CD4+ T cell counts and T cell activation levels were determined in fresh whole blood using CD3, CD4, CD8, and CD38 fluorochrome-labeled antibodies and a FACSCalibur flow cytometer (BD Biosciences) like previously reported [23]. In this article, the T cell activation status is determined by the percentage of CD38+ cells among the CD8+ T cells. Plasma and PBMC were separated from fresh whole blood, frozen down at −80°C and in liquid nitrogen, respectively, and shipped to Belgium. HIV and HSV-2 (Herpes simplex virus type 2) status were determined in plasma by ELISA. HIV-1 viral loads were quantified in plasma by the Amplicor HIV-1 Monitor assay, version 1.5 (Roche Diagnostics GmbH). PBMC were thawed, washed twice, and counted prior to mRNA and protein expression analysis.

### RNA extraction, DNase treatment and reverse transcription

PBMC samples were dissolved in TRIzol® Reagent (Invitrogen) and stored at −80°C for less than three months. Total RNA was isolated according to the manufacturer's instructions (Invitrogen). Removal of genomic DNA was performed with the DNA-free Kit (Ambion) following the manufacturer's protocol. Additional wash steps, once with 200 µl isopropanol (in the presence of 0.7 µl of glycogen) and twice with 75% ethanol, were performed to remove traces of contaminants. The NanoDrop ND-1000 spectrophotometer (Thermo Scientific) was used to quantify total RNA levels. Total DNase-treated RNA was stored at −80°C for less than one month. The iScript cDNA synthesis Kit (Bio-Rad Laboratories NV-SA) was applied to 400 ng of total DNase-treated RNA following the manufacturer's instructions. In parallel, 100 ng of the same RNA was dissolved in RNase/DNase-free water (no-RT control) at the same final concentration (20 ng/10.5 µl) as the RT (reverse transcribed) samples. Both RT and no-RT samples were stored at −80°C for less than one month. For each sample, the difference in quantification cycle (Cq)-value between RT and no-RT samples was higher than 10. During optimization of the qPCR protocol, RNA quality was evaluated by RNA gel electrophoresis (1% agarose gel). The 18S and 28S fragments were visualized with GelRed (VWR) versus a 1-kb DNA ladder (Invitrogen) and were observed in the expected 1∶2 ratio on representative samples.

### Real-time PCR

Messenger RNA (mRNA) expression levels were quantified by real-time PCR using the Bio-Rad CFX96 system and SYBR Green mix (Bio-Rad). Target-specific primers were designed using the Harvard website: http://pga.mgh.harvard.edu/primerbank/, except for *TRIM5α* and *TBP*, for which Primer3 software (v.0.4.0) was used. Specificity was tested using BLAST (NCBI). All primer pairs, except for *TRIM5*α, could be designed to span at least one intron. Name, GeneID, primer sequences, exon localization and amplicon length are listed in Table 1. The primer mixes were prepared by combining Forward (Fw) and Reverse (Re) Primers (Eurogentec), both at a concentration of 2 pmol/µl. Each optimized 25 µl reaction contained 12.5 µl of iQ SYBR Green Supermix (Bio-Rad), 2 µl of the primer mix and 5, 10 or 20 ng of the cDNA template at a volume of 10.5 µl. Each assay included, in triplicate, a standard curve, a no-template control (water), the RT samples, and, in the case of *TRIM5*α, the no-RT samples. The following subsequent heating steps were included as PCR cycling conditions: 50°C (10 min), 95°C (3 min), 40 cycles of 95°C (10 s) and 60°C (30 s) with fluorescence capturing following each cycle, 95°C (10 s), and melting curve acquisition with fluorescence capturing every 5 s during a temperature increase in 0.5°C increments from 65°C to 95°C. The amplicon length of the PCR product was validated by 2% agarose DNA gelelectrophoresis. PCR products were visualized with GelRed (VWR) versus a 100-bp DNA ladder (Invitrogen).

**Table 1:** Primers used for reverse transcriptase quantitative polymerase chain reaction (RT-qPCR).

	GeneID		Primer Sequence 5′ to 3′	Exon localization	Amplicon length
**Genes of interest**					
LEDGF/p75	11168	Fw*Re†	TCGACTTCAAAGGATACATGCTG GAGCTTGTTGCATTGTGACCT	1213	122
APOBEC3G	60489	FwRe	TCAGAGGACGGCATGAGACTT TGGAGCCTGGTTGCATAGAAA	55 and 6	107
TRIM5alpha	85363	FwRe	TGCTGGCTTCCAACCTGAT ACAGAGAGGGGCACAATGAA	88	165
Tetherin (BST2)	684	FwRe	GAGTGTCGCAATGTCACCCAT GGAAGCCATTAGGGCCATCAC	11 and 2	120
**Reference genes**					
UBC‡	7316	FwRe	ATTTGGGTCGCAGTTCTTG TGCCTTGACATTCTCGATGGT	12	123
B2M§	567	FwRe	GGCTATCCAGCGTACTCCAAA CGGCAGGCATACTCATCTTTTT	1 and 22	246
GAPDH∥	2597	FwRe	TGTTGCCATCAATGACCCCTT CTCCACGACGTACTCAGCG	35	202
RPL13A¶	23521	FwRe	CGAGGTTGGCTGGAAGTACC CTTCTCGGCCTGTTTCCGTAG	78	121
TBP#	6908	FwRe	ACCCAGCAGCATCACTGTTT CCAAGCCCTGAGCGTAAG	1 and 22	200

* Forward primer,

† Reverse primer,

‡ Ubiquitin C,

§ Beta-2-Microglobulin,

∥ Glyceraldehyde 3-phosphate dehydrogenase,

¶ 60S ribosomal protein L13a, and

# TATA-binding protein.

### mRNA data analysis

The obtained results (Cq-values) were exported from the Bio-Rad CFX Manager version 1.5 and imported into qbase^PLUS^ software, version 1.5, from BioGazelle (Ghent, Belgium) following baseline subtraction and manual threshold matching. The GeNorm applet selected the reference genes *B2M* and *GAPDH* out of five genes (*UBC, B2M, GAPDH, RPL13A, TBP*) as the most stably expressed across the different study populations (25 samples, all applied in triplicate). Both reference genes were used to normalize target gene expression in the qbase^PLUS^ software. For the standard curve, a 2-fold serial dilution of cDNA of one Caucasian healthy control was used. Cq-values were converted into relative expression values taking into account amplification efficiencies, inter-run variations, and normalization factors. Outliers were excluded when a difference of more than 1.0 was registered in the Cq-values. Finally, CNRQ (Calibrated Normalized Relative Quantity) values were exported from the qbase^PLUS^ software and statistically investigated.

### Intracellular and surface protein staining

The protein expression levels of LEDGF/p75, APOBEC3G, TRIM5α, and tetherin were investigated in CD4+ T cell and CD14+ monocyte subsets based on simultaneous intracellular and/or cell surface staining of PBMC. The following reagents were used: 0.1% bovine serum albumin (Acros Organics) and 0.05% sodium azide (Merck) in phosphate buffered saline (PBS, Lonza) as washing buffer; Reagents A and B to fix and permeabilize cells, respectively (Leucoperm kit, AbD Serotec); primary mouse unlabeled antibody (isotype IgG1 and IgG2a) and anti-LEDGF/p75 (IgG1) from BD Biosciences, anti-APOBEC3G and anti-TRIM5α (both IgG1) from the AIDS Research and Reference Reagent Program (Division of AIDS, NIAID, NIH), and anti-tetherin (IgG2a) as kindly provided by Chugai Pharmaceutical Co., Japan; secondary FITC-labeled antibody (BD Biosciences); normal mouse serum (eBioscience); membrane antibodies anti-CD4-PerCP, anti-CD45RO-APC, anti-CD38-PE, anti-CD14-APC, and anti-CD16-PE from BD Biosciences; and 1% paraformaldehyde (Sigma-Aldrich). LEDGF/p75, APOBEC3G, TRIM5α, and tetherin were analyzed using an in-house optimized intracellular staining (ICS) protocol [24], [25]. Cells were washed (630×*g*, 10 min, RT), fixed (10 min, RT), washed twice, permeabilized (30 min, 4°C), washed, incubated with primary antibody (24 h, 4°C), washed twice, incubated with secondary antibody supplemented with reagent B (30 min, 4°C), washed twice, incubated with normal mouse serum (10 min, 4°C), incubated with a membrane antibody cocktail (CD4/CD45RO/CD38 or CD4/CD14/CD16 to study lymphocytes or monocytes, respectively, 20 min, 4°C), washed twice, and dissolved in 1% paraformaldehyde prior to data acquisition (FACSCalibur flow cytometer, BD Biosciences). To study membrane tetherin levels, PBMC were processed according to the protocol above with a 30-min anti-tetherin incubation at 4°C, without fixation or permeabilization steps. In general, at least 25000 and 50000 events were respectively measured to study expression patterns in CD4+ lymphocytes and CD14+ monocytes. Data analysis with CellQuest Pro Software (BD Biosciences) produced median fluorescence intensity (MFI) values, reflecting the median amount of bound antibody per cell and thus, the protein's abundance. Host protein MFI-values were corrected for non-specific background staining by subtracting isotype MFI-values.

### Statistical analyses

Differences in expression levels between individuals of different study groups were investigated with non-parametric Mann-Whitney *U* tests. Correlation analyses were performed using non-parametric Spearman's rank correlation tests. Observations were considered statistically significant when p<0.05. Statistical analyses were performed with SPSS version 17.0, and graphs were drawn with GraphPad Prism, version 5.

## Results

### Study population

Twenty three HIV-1-exposed seronegative subjects (HESN), 23 healthy controls (HC), and 45 HIV-1-infected patients were studied. Nine HIV-1-infected patients were untreated (HIV-UT) and 36 were antiretroviral therapy-treated (HIV-ART). Several parameters like age, gender, duration of the sexual relationship, CD4 count, number of sexual contacts per month, condom use, and HSV-2 serostatus were compared between HESN and HC, HIV-UT and HC, and HIV-ART and HIV-UT (Table 2). The HC subjects were in a monogamous relationship for at least 7.5 years at the time of enrollment. They reported a significantly lower percentage of condom use. HIV-1-infected subjects were more often male individuals. Therapy-naïve HIV-1 infected subjects (HIV-UT) showed significantly lower CD4 count levels than HC, and a higher prevalence of HSV-2 seropositivity.

**Table 2:** Characteristics of HIV-exposed seronegative, HIV-1-infected, and healthy control subjects included in the study.

	HC*(n = 23)	HESN†(n = 23)	HIV-UT‡(n = 9)	HIV-ART§(n = 36)	p-value		
					HESN vs. HC	HIV-UT vs. HC	HIV-ART vs. HIV-UT
Age, years	43(37–46)	41(33–47)	46(37–51)	44(36–51)	0.409	0.449	0.955
Gender (% male)	47.8	43.5	88.9	72.2	0.770	**0.036**	0.303
CD4 count (cells/µl)	789(703–1090)	738(548–891)	228(133–337)	334(195–412)	0.215	**<0.001**	0.294
Number of sexual contacts per month	8(3–12)	5(4–8)	5(4–11)	7(4–8)	0.164	0.831	0.831
Duration of sexual relation, years	12(8–19)	11(8–18)	11(9–18)	10(8–16)	0.938	0.866	0.540
Condom use (% always)	0	61	44	50	**<0.001**	**0.001**	0.768
HSV-2 serostatus (% HSV-2 positive)	22	45	78	59	0.095	**0.004**	0.301

Data are median (interquartile range) values or percentages if indicated.

* Healthy controls,

† HIV-exposed seronegatives,

‡ therapy-naïve HIV-1-infected subjects,

§ antiretroviral therapy-treated HIV-1-infected subjects. P-values (Mann Whitney *U*) below 0.05 are in bold.

### Differences in mRNA expression in PBMC

The mRNA expression levels of LEDGF/p75, APOBEC3G, TRIM5α, and tetherin were analyzed for all study subjects in PBMC by real-time qPCR (Table 3). None of the genes displayed a significant difference in mRNA expression between HESN and HC. HIV-UT showed significantly lower levels of LEDGF/p75 (p = 0.008) and higher levels of tetherin (p = 0.027) than HC. Additionally, higher expression levels of APOBEC3G (p = 0.078), TRIM5α (p = 0.016), and tetherin (p<0.001) were observed in HIV-UT relative to HIV-ART subjects.

**Table 3:** Expression levels of LEDGF/p75, APOBEC3G, TRIM5α, and tetherin in PBMC of HIV-exposed seronegative, HIV-infected, and healthy control subjects.

	HC*	HESN†	HIV-UT‡	HIV-ART§	p-value		
					HESN vs. HC	HIV-UT vs. HC	HIV-ART vs. HIV-UT
**mRNA expression**	N = 20	N = 20	N = 9	N = 31			
LEDGF/p75	1.22(0.88–1.50)	1.03(0.83–1.36)	0.72(0.53–1.11)	0.92(0.70–1.10)	0.387	**0.008**	0.391
APOBEC3G	0.98(0.79–1.23)	0.99(0.86–1.25)	1.29(1.00–1.38)	0.96(0.83–1.22)	1	0.172	0.078
TRIM5α	1.08(0.79–1.42)	0.85(0.74–1.27)	1.22(0.92–1.89)	0.78(0.65–1.18)	0.372	0.370	**0.016**
Tetherin	1.12(0.88–1.36)	1.10(0.96–1.29)	1.46(1.14–1.85)	0.99(0.78–1.06)	1	**0.027**	**<0.001**
**Protein expression**	N = 23	N = 23	N = 9	N = 36			
**CD4+ lymphocytes**							
LEDGF/p75	113.10(97.62–126.05)	98.80(77.95–110.13)	121.91(85.58–53.40)	106.85(94.77–122.80)	**0.022**	0.516	0.371
APOBEC3G	39.61(35.48–42.93)	38.01(31.08–44.88)	39.68(36.58–47.88)	40.64(35.53–47.08)	0.613	0.572	0.691
TRIM5α	107.57(96.87–123.01)	112.80(93.67–125.22)	126.13(100.47–156.08)	115.15(100.26–134.41)	0.939	0.148	0.427
Membrane tetherin	4.16(3.71–4.68)	4.35(3.94–4.81)	6.55(5.34–9.29)	4.77(4.03–5.19)	0.733	**<0.001**	**<0.001**
Total tetherin	5.32(4.64–6.74)	5.33(4.43–6.54)	10.73(7.20–18.52)	5.88(5.43–6.90)	0.809	**0.003**	**0.004**
**CD14+ monocytes**							
LEDGF/p75	49.26(40.55–70.39)	45.51(36.73–71.27)	77.05(62.22–86.85)	45.65(40.94–59.45)	0.750	**0.005**	**0.002**
APOBEC3G	102.09(81.37–128.12)	98.62(80.19–13.40)	123.28(111.52–172.55)	105.30(90.89–116.28)	0.328	**0.031**	**0.002**
TRIM5α	280.38(234.04–335.57)	283.79(235.40–332.42)	398.98(317.44–513.08)	287.94(257.72–331.20)	0.869	**0.005**	**0.002**
Membrane tetherin	1.62(0.00–6.67)	2.42(0.00–4.95)	11.77(6.38–21.86)	4.78(1.19–7.17)	0.894	**0.002**	**0.005**
Total tetherin	49.28(40.00–65.42)	49.84(40.99–55.70)	79.48(59.27–113.97)	46.13(38.27–50.87)	0.956	**0.003**	**<0.001**

Data are median (interquartile range) values depicting Calibrated Normalized Relative Quantity (CNRQ-)values for mRNA and Median Fluorescence Intensity (MFI-)values for protein expression.

* Healthy controls,

† HIV-exposed seronegatives,

‡ therapy-naïve HIV-1-infected subjects,

§ antiretroviral therapy-treated HIV-1-infected subjects. P-values (Mann Whitney *U*) below 0.05 are in bold. Of note, due to sample loss during RNA extraction, data on mRNA expression were gathered for slightly less study subjects.

### Differences in protein expression in lymphocytes and monocytes

Next, we analyzed protein expression levels of LEDGF/p75, APOBEC3G, TRIM5α, and tetherin in CD4+ lymphocytes and CD14+ monocytes by a novel intracellular staining assay [25]. Expression of LEDGF/p75 was found to be significantly reduced (p = 0.022) in CD4+ lymphocytes of HESN compared to HC (Table 3). APOBEC3G, TRIM5α, and tetherin protein levels were similar for HESN and HC. HIV-UT showed significantly higher tetherin levels in CD4+ lymphocytes and significantly higher levels of all four proteins in CD14+ monocytes compared to HC and HIV-ART.

### Correlations between mRNA and protein levels

**Figure 1:**
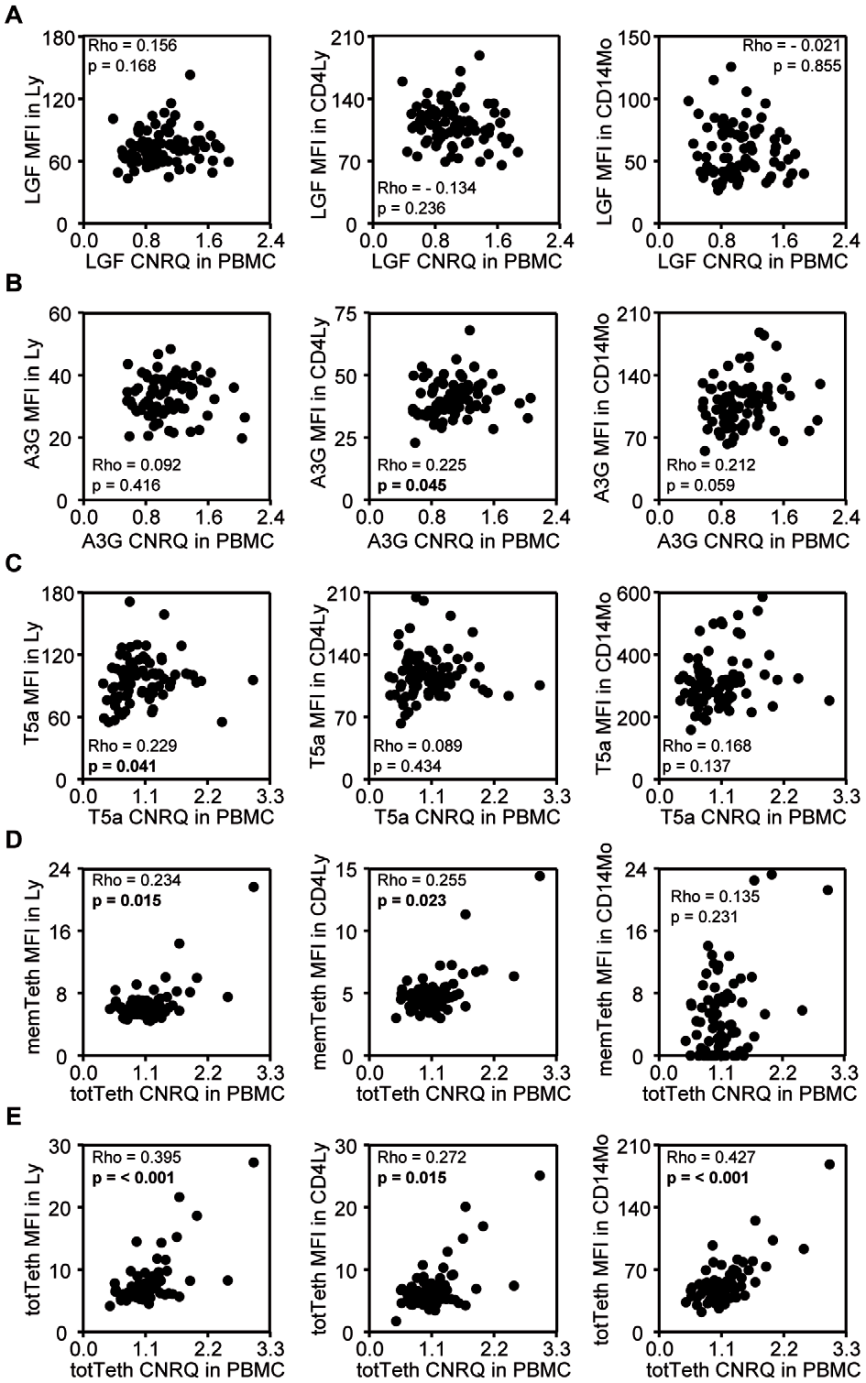
Correlations between mRNA and protein levels. Correlation patterns were studied in all study subjects between mRNA (CNRQ-values, x-axis) and protein (MFI-values, y-axis) levels of (A) LEDGF/p75 (LGF), (B) APOBEC3G (A3G), (C) TRIM5α (T5a), (D) membrane tetherin (memTeth), and (E) total tetherin (totTeth). mRNA levels were studied in PBMC while protein levels were analyzed in lymphocytes (Ly, left), CD4+ lymphocytes (CD4Ly, middle), and CD14+ monocytes (CD14Mo, right). CNRQ-values refer to Calibrated Normalized Relative Quantity of mRNA expression levels and MFI-values refer to Median Fluorescence Intensity of protein expression levels. Spearman's Rho (Rho) and p-values were obtained by non-parametric Spearman's rank correlation tests. P-values below 0.05 are in bold.

Subsequently, we correlated mRNA levels in PBMC with protein levels specifically in lymphocytes and monocytes within the entire study population including HESN, HC, HIV-UT, and HIV-ART subjects (Figure 1). For most HIV-related host factors, we observed a significant positive correlation between mRNA and protein level as was most prominent for total tetherin, whereas no significant correlations were observed for LEDGF/p75.

### Correlates of reduced LEDGF/p75 in HESN

To obtain insight into the possible role played by LEDGF/p75 in resistance to HIV-1 infection, we studied the reduced LEDGF/p75 expression levels in HESN considering the duration of the sexual relation, the number of sexual contacts per month, the frequency of condom use, and the HSV-2 serostatus (Table 4). We found that LEDGF/p75 expression levels were most reduced among HESN with the highest number of sexual contacts per month (p = 0.065), whereas no difference in LEDGF/p75 expression was found in the HIV-1-negative control group (Table 4).

**Table 4:** Expression levels of LEDGF/p75 versus HIV-1 risk/exposure parameters within different study populations of interest.

	HC*			HESN†		
	subgroup 1^a^	subgroup 2^b^	P-value	subgroup 1	subgroup 2	P-value
Sexual contacts/month(<^a^ vs ≥^b^ median value)	109.47(98.59–124.73)	114.26(94.84–137.76)	0.537	105.63(95.04–112.09)	85.37(74.88–104.96)	0.065
Duration of relation, years(<^a^ vs ≥^b^ median value)	113.10(99.57–141.30)	110.90(89.88–124.04)	0.284	104.58(73.50–111.30)	95.46(81.66–112.71)	1.000
Condom use(always^a^ vs not always^b^)	-	110.23(96.24–126.13)	ND	98.30(69.33–111.37)	98.80(81.66–112.66)	0.571
HSV-2 status(negative^a^ vs positive^b^)	110.23(95.70–124.73)	123.96(96.80–132.58)	0.502	98.30(76.20–110.11)	101.50(85.31–126.40)	0.468

* Healthy controls,

† HIV-exposed seronegatives. Each HIV-1 risk/exposure parameter (left column) was used to split the study population in two subgroups (^a^ subgroup 1 versus ^b^ subgroup 2) based on a categorical or on a numerical parameter. Numerical parameters use median values (see Table 2) to split the study group in two subgroups comparable in size. LEDGF/p75 expression levels are median (interquartile range) values. Within each study population, the P-value (Mann Whitney *U*) was calculated for each HIV-1 risk/exposure parameter. ND: not determined as none of the subjects used a condom.

### Correlations between expression levels and T cell activation

**Figure 2:**
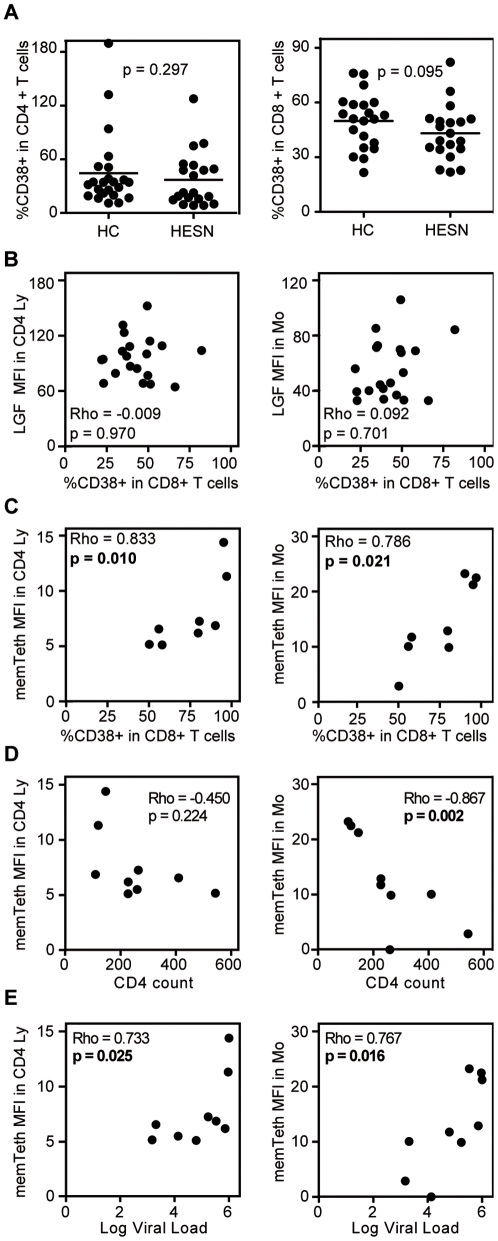
Statistical analyses within HESN and untreated HIV-1-infected subjects. (A) Difference in CD4 (left) and CD8 (right) T cell activation status (y-axis) between HESN and HC (x-axis). P-values were obtained through the Mann Whitney *U* test. (B) Correlation pattern in HESN subjects between LEDGF/p75 protein levels (LGF, y-axis) in CD4+ lymphocytes (CD4 Ly, left) and CD14+ monocytes (Mo, right) and T cell activation status (x-axis). (C–E) The graphs depict correlation patterns in CD4+ lymphocytes (CD4 Ly, left) and CD14+ monocytes (Mo, right) of HIV-UT subjects between membrane tetherin protein levels (memTeth, y-axis) and T cell activation status (x-axis, C), CD4 count (cells/mm^3^) (x-axis, D) and log viral load (x-axis, E). The subject's general T cell activation status is described by the percentage of CD38+ cells among the CD8+ T cells (B,C). Spearman's Rho (Rho) and p-values were obtained by non-parametric Spearman's rank correlation tests. P-values below 0.05 are in bold. MFI = Median Fluorescence Intensity.

Finally, we investigated whether the observed differences in mRNA and/or protein expression level could be correlated to T cell activation levels and/or disease status (viral load and CD4 count). Whereas no significant difference was observed in CD4+ T cell activation level (% CD38+ cells among the CD4+ T cells, p = 0.297), HESN showed a trend towards lower CD8+ T cell activation levels than HC (p = 0.095) in Figure 2A. Reduced T cell activation levels among HESN did not correlate with their reduced LEDGF/p75 protein expression levels (p = 0.970, Figure 2B). Neither did we observe differences in LEDGF/p75 expression between distinct CD4+ lymphocytes based on CD45RO (memory) or CD38 (activation) staining among HESN (data not shown). In HIV-UT, T cell activation levels correlated directly with TRIM5α (p = 0.002) and tetherin (p = 0.086) mRNA levels, and inversely with LEDGF/p75 mRNA levels (p = 0.037) (data not shown). Membrane and total expression levels of tetherin, in particular, correlated directly with T cell activation and the viral load, and inversely with the CD4 count. These correlations were most prominent for membrane tetherin as is depicted in Figure 2C–E.

## Discussion

In this study, we explored whether the expression of LEDGF/p75, APOBEC3G, TRIM5α, and tetherin could play a role in HIV-1 resistance. We studied mRNA levels in PBMC and protein levels in CD4+ lymphocyte and monocyte subsets. We observed significantly reduced levels of LEDGF/p75 protein expression in the CD4+ T cells of HESN compared to those of HC, whereas similar mRNA levels were found for both HESN and HC. Our findings of lower levels of LEDGF/p75 in CD4+ lymphocytes of HESN are in line with two recent HESN-studies showing reduced gene expression levels of several host genes important for HIV replication [26], [27]. Lower LEDGF/p75 protein levels may cause HIV-1 integration to occur less efficiently, thus contributing to HIV-1 resistance in HESN subjects. This hypothesis is supported by an *in vitro* experiment that found a direct correlation between LEDGF/p75 expression levels and the efficiency of HIV-1 integration in the host genome [28]. Other studies, however, reported that efficient knockdown or knock-out of LEDGF/p75 is not sufficient to completely abolish HIV-1 replication [29]–[31], suggesting that other factors may be needed in concert with LEDGF/p75 to limit HIV integration in HESN subjects.

We observed no differences in mRNA or protein levels of APOBEC3G, TRIM5α, or tetherin between HESN and HC. These findings are at variance with previous reports showing elevated APOBEC3G expression levels in HESN compared with HC [16], [21]. On the other hand, it has been suggested that reduced exposure to HIV-1 results in comparable APOBEC3G mRNA levels for HESN and HC over time [16]. In our HESN population, most HIV-1-infected partners were treated with antiretroviral therapy, and all couples received counseling to promote condom use. Therefore, the lack of distinct expression patterns may indeed result from the relatively low levels of HIV exposure in our population. Moreover, Reddy et al [19] observed similar levels of APOBEC3G mRNA between pre-infection values of seroconverters and exposed persistently negative individuals, suggesting that APOBEC3G mRNA levels would not contribute to protection against HIV-1.

Untreated HIV-1-infected patients (HIV-UT) showed significantly lower LEDGF/p75 and higher tetherin mRNA levels than HC, and higher APOBEC3G, TRIM5α, and tetherin mRNA levels than HIV-ART. At protein level, HIV-UT expressed significantly enhanced levels of tetherin in the lymphocytes and of all four proteins in the monocytes. These data suggest that HIV-1-infected subjects try to curb HIV-1 replication by increasing the expression level of the antiviral restriction factors.

Interestingly, increased levels of tetherin in HIV-UT correlated directly with T cell activation and the viral load and inversely with the CD4 count. This finding suggests that tetherin is not capable of controlling viral replication; instead it appears to be a marker of advanced HIV disease in therapy-naïve HIV-1-infected patients. Recently, Coleman et al [32] also reported increased tetherin expression levels upon exposure to HIV-1 without it being capable of restricting cell-to-cell spread of the virus. At least two other studies support our observations by suggesting that the tetherin-induced accumulation of unreleased virions at the cell membrane could actually promote cell-to-cell spread, e.g., by enhancing fusogenicity or regulating the integrity of the viral synapse [33], [34]. In turn, accumulation of virus particles at the cell membrane may trigger further increases in immune activation [35], [36]. Thus, tetherin may have a dual role during HIV-1 infection and disease. As an innate mediator, tetherin may be involved in the control of early or acute virus replication but it could become ineffective as the infection progresses.

T-cell activation levels also correlated directly with mRNA levels of TRIM5α but not with those of APOBEC3G; nevertheless we expect that similar mechanisms like those observed for tetherin are involved here. Indeed, type I interferons which are induced upon viral replication are known to upregulate APOBEC3G [15], [37]–[39], TRIM5α [40], [41], and tetherin [36], [42]. High-level interferon receptor expression on monocytes [21] may explain why our data showed relatively higher increases of APOBEC3G, TRIM5α, and tetherin in monocytes than in lymphocytes.

Intriguingly, HIV-UT expressed reduced mRNA levels in PBMC and elevated protein levels in monocytes for LEDGF/p75, relative to HC. The mRNA levels correlated inversely (p = 0.05; Rho = −0.667) and the protein levels tended to correlate directly (p = 0.170; Rho = 0.5) with the viral load of these patients (data not shown). These correlations suggest that LEDGF/p75 expression may also account for disease progression in HIV-UT, supporting recently described observations by Madlala et al [27]. Parallel to type I interferon induction, viral replication induces the production of several cytokines and chemokines amongst which Tumor Necrosis Factor alpha (TNF-α), which can induce LEDGF/p75 levels [43], [44]. Of note, long period or high level exposure to TNF-α makes LEDGF/p75 levels decrease again [44], which may play a role in the difference seen between LEDGF/p75 mRNA and protein levels.

Apart from the reasoning in the previous paragraph, mRNA and protein levels may differ as mRNA is not necessarily translated into (functional) protein [45], and external factors may act differently upon protein and mRNA levels [39], [46]. Our data e.g. also show a difference in mRNA versus protein expression for LEDGF/p75 in HESN (Figure 1A). Nevertheless, we did observe some correlations between protein levels in (CD4+) lymphocytes, and mRNA levels in PBMC which are predominantly composed of lymphocytes (Figure 1B–E). Of note, due to the limited number of CD4+ cells in HIV-1-infected subjects, we were obliged to work with PBMC for quantification of mRNA levels. On the other hand, the flow cytometry based method allowed us to investigate protein levels in PBMC subsets. As earlier studies also focused on mRNA levels in PBMC, we could easily compare our results with these results previously obtained. For example, our data supported that APOBEC3G mRNA levels are similar for HESN and HC [19]. On the other hand, in literature, limited information is forehand regarding protein expression in particular cell-types while proteins are considered to be of greater relevance in the “direct” fight against HIV-1 infection as they are the “work horses” of the cell.

In summary, we observed significantly lower levels of LEDGF/p75 in CD4+ lymphocytes of HESN subjects, suggesting that LEDGF/p75 may play a role in the *in vivo* resistance to HIV-1 infection. Untreated HIV-1-infected patients showed increased levels of the antiviral proteins APOBEC3G, TRIM5α, and tetherin, with tetherin in particular as a possible marker of advanced HIV disease. Future studies of LEDGF/p75 expression in other populations of HESN subject will be required to confirm our findings. Understanding the role of HIV-1-related host factors in HIV-1 susceptibility and disease could be of great interest for the development of future antiviral therapies.
